# Abundant and equipotent founder cells establish and maintain acute lymphoblastic leukaemia

**DOI:** 10.1038/leu.2017.140

**Published:** 2017-05-26

**Authors:** A Elder, S Bomken, I Wilson, H J Blair, S Cockell, F Ponthan, K Dormon, D Pal, O Heidenreich, J Vormoor

**Affiliations:** 1 Wolfson Childhood Cancer Research Centre, Northern Institute for Cancer Research, Newcastle University, Newcastle upon Tyne, UK; 2 Department of Paediatric and Adolescent Haematology and Oncology, Great North Children’s Hospital, Newcastle upon Tyne Hospitals NHS Foundation Trust, Newcastle upon Tyne, UK; 3 Institute of Genetic Medicine, Newcastle University, Newcastle upon Tyne, UK; 4 Bioinformatics Support Unit, Newcastle University, Newcastle upon Tyne, UK

## Abstract

High frequencies of blasts in primary acute lymphoblastic leukaemia (ALL) samples have the potential to induce leukaemia and to engraft mice. However, it is unclear how individual ALL cells each contribute to drive leukaemic development in a bulk transplant and the extent to which these blasts vary functionally. We used cellular barcoding as a fate mapping tool to track primograft ALL blasts *in vivo*. Our results show that high numbers of ALL founder cells contribute at similar frequencies to leukaemic propagation over serial transplants, without any clear evidence of clonal succession. These founder cells also exhibit equal capacity to home and engraft to different organs, although stochastic processes may alter the composition in restrictive niches. Our findings enhance the stochastic stem cell model of ALL by demonstrating equal functional abilities of singular ALL blasts and show that successful treatment strategies must eradicate the entire leukaemic cell population.

## Introduction

In recent years, there has been increasing focus on the extent to which intra-tumour heterogeneity influences the development and evolution of cancer.^1^ There is substantial evidence that ALL samples consist of a genetically heterogeneous subclonal architecture, with pools of subclones related by Darwinian style ancestral trees, and that this diversity can be maintained following xenotransplantation.^2, 3, 4, 5, 6, 7^ These data support a clonal evolution model whereby individual clones acquire fitness-modulating mutations, resulting in constant changes to the composition of the propagating cell population under selection pressure. This has important clinical implications, as relapse clones often descend from minor diagnostic or pre-leukaemic clones, which evolve during disease progression and become enriched through selection in response to therapy.^8, 9^ Many of these studies into tumour heterogeneity have looked at a limited subset of genetic markers, so may not reflect the true complexity of ALL at the single-cell level. In addition, these genetically distinct subclones will consist of a multitude of individual cells which may not contribute equally to disease progression. Our previous results using limiting dilution analysis demonstrate that B-ALL cells able to engraft immunodeficient mice are common and not restricted to populations of specific immunophenotypes.^10, 11^ This is consistent with a stochastic model of engraftment, whereby most cells are able to propagate the tumour, as opposed to a rare stem cell hierarchy. However, previous studies have predominantly assessed engraftment potential as opposed to fate, so there are several unanswered questions as to how individual propagating cells actually collaborate to drive the disease. Although many blasts have the potential to propagate the disease, is the leukaemia actually driven by a more limited subset of the bulk population? Do the engrafting cells differ functionally? Is there evidence of clonal dormancy or clonal succession over serial transplants? In addition, it is not known how the composition of leukaemias may vary at different sites. Insights from solid tumours have clearly demonstrated spatial heterogeneity within tumours.^12^ It is generally assumed that leukaemias will be more homogeneous in different parts of the body; however, this has not been studied in detail.

We addressed these questions using cellular barcoding to label high-risk patient-derived ALL cells with unique, heritable DNA markers.^13, 14^ This approach allows us to examine cellular behaviour at the level of individual engrafting blasts as opposed to bulk populations. Our results demonstrate that the cells which drive ALL in xenotransplanted mice are both abundant and functionally equipotent, with every engrafting cell having equal capability to propagate the leukaemia. Despite this, the composition of ALL can vary stochastically in different parts of the bone marrow, suggesting that sampling the leukaemia from a single site may not be representative of the whole disease.

## Materials and methods

See Supplementary Materials for detailed methods.

### Barcoding of primograft ALL samples

Oligonucleotides containing the random barcode sequence were annealed and cloned into a lentiviral vector, pSLIEW.^15^ The complexity of the library was validated by Illumina MiSeq sequencing. Virus production and transduction of primograft material was performed as described previously.^15^ Cells were injected into the left femurs of NSG mice. Engraftment was monitored using the IVIS Spectrum *In Vivo* Imaging System (Caliper Life Sciences) and mice were kept until they began to exhibit clinical symptoms necessitating humane killing. Samples were collected from the spleen, femurs, tibias and meninges.

### Sequencing and data analysis

Barcode regions were amplified from genomic DNA using primers specific to the barcode region and sequenced using an Illumina MiSeq. Sequencing data was analysed using a python script (provided in Supplementary Materials) to extract barcode sequences and frequencies. Barcodes with the lowest read counts were removed until all remaining comprised greater than 0.01% of the total reads. Barcodes above this level were manually removed if they had single base changes from high frequency barcodes in the same sample, or were identical to higher frequency barcodes from other mouse samples in the same sequencing run (see Supplementary Methods for further detail).

### Measurement of gene diversity index (G)

R code for the estimation of G is at the web site https://github.com/ijwilson/diversit-tag.

## Results

### ALL founder cells are abundant and contribute equally to the leukaemia

**Figure 1:**
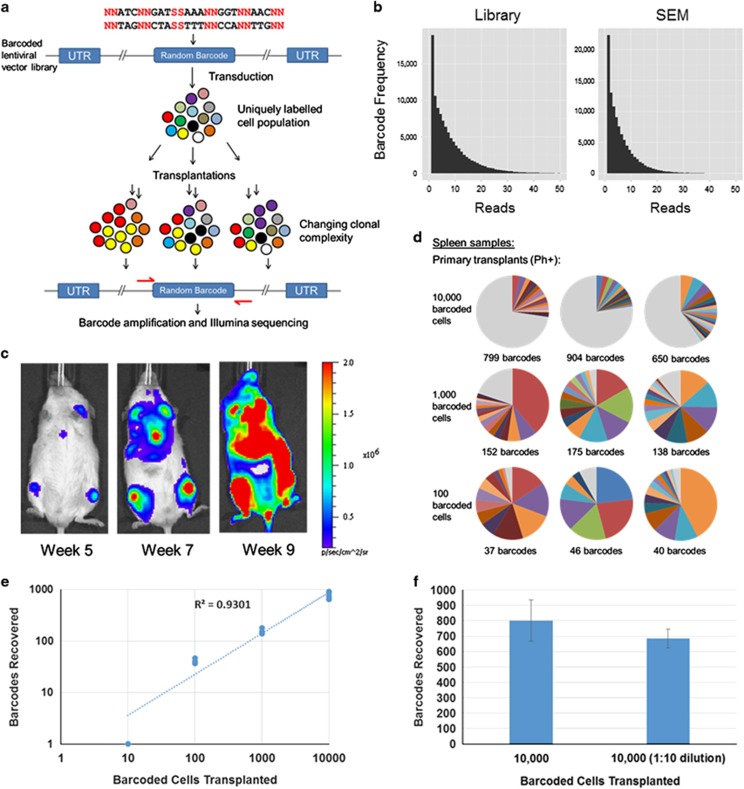
ALL founder cells have equal functional potential. (**a**) Schematic of experimental design. (**b**) Distribution of sequencing reads from barcode plasmid library (left) and SEM cells transduced with barcode library (right). (**c**) IVIS images showing leukaemic development of barcoded Ph+ (L4951) sample following intrafemoral transplant. Ten thousand barcoded cells were transplanted. (**d**) Composition of barcoded population at varying transplant doses for Ph+ (L4951) ALL sample. All transplant numbers represent the number of barcoded cells transplanted, which comprised ~10% of the total transplanted population. Each pie chart shows barcode composition in spleen sample from a single mouse. Coloured segments each represent a unique barcode comprising >1% of the total, light grey segment shows all other barcodes <1%. Colours do not represent the exact same barcode on different pie charts. (**e**) Graph showing number of barcodes recovered from Ph+ (L4951) spleen samples at different transplant doses. Each point is a single mouse spleen. (**f**) Graph showing barcodes recovered from Ph+ (L4951) spleen following transplant of cell populations containing 10 000 barcoded cells (*n*=3) or 10 000 barcoded cells further diluted 1:10 with unlabelled cells (*n*=3). *P*=0.174 using two-tailed student’s *t*-test, error bars show standard deviation.

B-ALLs contain high frequencies of cells capable of engrafting and propagating leukaemia in immunodeficient mice.^10, 11^ However, these studies purely detail the engraftment potential of B-ALL cells and do not provide information about the number of founder cells that actually engraft in bulk transplants and their subsequent fate. To allow us to map the fate of individual blasts in leukaemia development, we developed a cellular barcoding method to label individual cells with unique markers (Figure 1a). We cloned a random barcode library based on a previously published design^13^ into a lentiviral vector,^15^ achieving a complexity of over 100 000 unique barcodes with unbiased composition, which could be maintained following transduction of the SEM cell line (Figure 1b). NOD.Cg-*Prkdc*^*scid*^ *Il2rg*^*tm1Wjl*^/SzJ (NSG) mice were transplanted with varying doses of four barcoded high-risk ALL primograft samples and engraftment monitored using IVIS imaging (Figure 1c and Table 1). To reduce the risk of multiple lentiviral integrations, we limited transduction levels to ~10% based on green fluorescent protein (GFP) expression^16^ (Supplementary Figure S1a). Our first question was whether blasts able to engraft and establish the leukaemia in mice (founder cells) differ in terms of their ability to contribute to the primary leukaemia and to self-renew over serial transplants. To look for evidence of this, we first examined the distribution of recovered barcodes in primary spleen samples. When we transplanted high numbers (>5000) of labelled cells, spleen samples from two different patient samples had substantial diversity, with no single barcode representing more than 5% of the total population (Figure 1d, top panel and Supplementary Figure S1b). This suggests a high degree of functional homogeneity among ALL cells, with the leukaemia driven by many barcoded cells contributing at similar frequencies, as opposed to dominance by a few clones. To further support this hypothesis, we transplanted limiting dilutions of cells. As expected, this led to a reduced overall complexity, although these spleens still typically contained 10–20 dominant barcodes at comparable frequencies as opposed to a single dominant clone (Figure 1d, lower panels). We calculated engrafting cell frequencies in the spleens of primary transplants as between 1 in 29 and 1 in 2 across all four samples (Table 1), based on a barcode detection threshold of 0.01% (see ‘Materials and Methods’ section). This is in line with our previous estimates of cells with engraftment potential, and demonstrates that this potential is translated into a high number of founder cells contributing to the leukaemia. The number of barcodes recovered increased linearly with the number of cells transplanted (Figure 1e), suggesting we had not yet reached a saturation point which limited the maximum number of cells able to engraft. In support of this, diluting the barcoded cells 1:10 by addition of an extra 900 000 untransduced cells did not substantially alter the engraftment frequency (Table 1 and Figure 1f) or the leukaemic composition of the spleen (Supplementary Figure S1c), demonstrating that the presence of large numbers of unlabelled cells does not affect engraftment of the labelled population.

**Figure 2:**
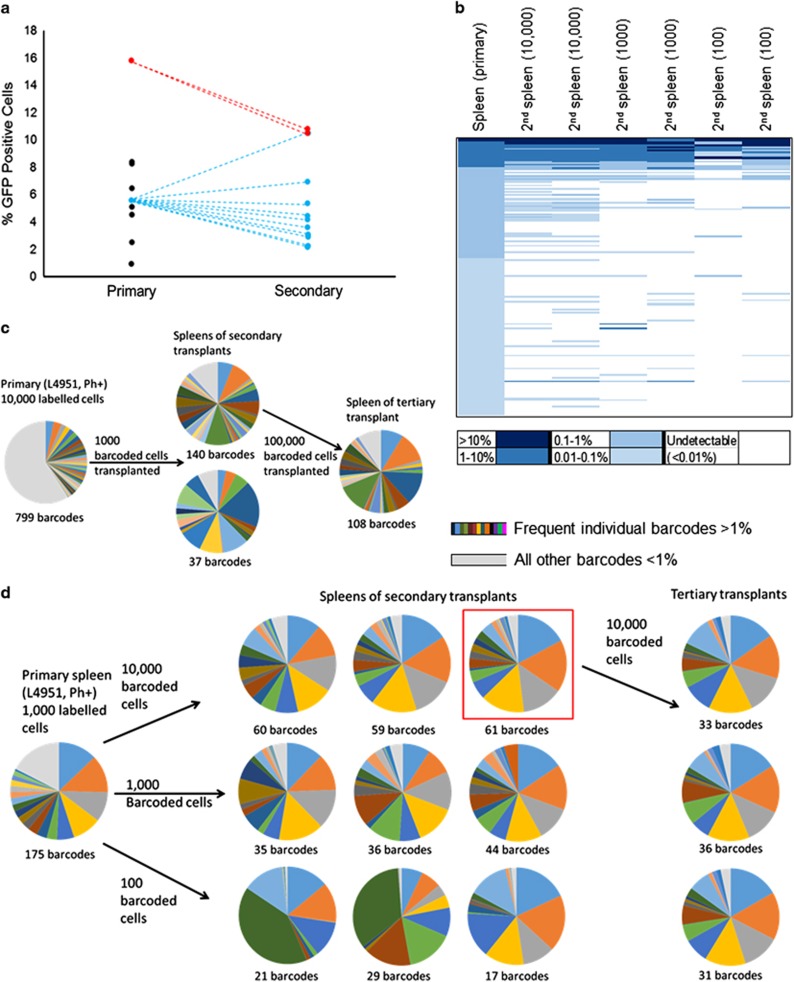
ALL founder cells maintain self-renewal capacity over serial transplants. (**a**) Change in frequency of GFP+ cells in spleen samples taken from primary and secondary transplants. Each point represents a single mouse, dashed lines link primary sample to corresponding secondary recipients. (**b**) Heat map showing frequency of individual barcodes in primary spleen sample (left hand column) compared to secondary recipients for Ph+ (L4951) sample. Numbers in brackets show barcoded cells transplanted. Each horizontal band represents a single barcode. Barcodes at frequencies below 0.01% of the total were considered undetectable in our experimental system. (**c**, **d**) Barcode composition in spleens of secondary and tertiary transplants compared to primary sample. Primary samples were spleens from initial transplants of 10 000 (**c**) or 1000 (**d**) barcoded cells. Numbers below each chart represent the total number of recovered barcodes in that sample, which were also present in the parent sample. Colours correspond to the same barcode for each mouse within each transplant set (for example, within **c**), but not between (for example, **c** compared to **d**).

Selected spleen samples were re-transplanted into secondary and then tertiary recipients to assess self-renewal. The percentage of GFP-expressing cells fluctuated following secondary transplants but did not show any consistent change between samples (Figure 2a), showing that the transduction did not substantially affect engraftment capability. Secondary recipients showed a reduction in absolute barcode numbers compared to primary, however, this was due to loss of rare (<0.5%) barcodes in primary samples (Figure 2b) resulting from transplantation of cell numbers too low to preserve the full repertoire of rarer barcodes (low coverage). Across all samples, all barcodes (57/57) with a frequency above 1% in the primary sample were detected in secondary transplants. The spleens of secondary and tertiary recipients exhibited a similar barcode complexity to that of the parent sample, with substantial variability in the composition only occurring at transplant doses lower than the complexity of the original sample (Figures 2c and d and Supplementary Figure S2). We also did not observe any minor barcodes in primary transplants, which became dominant in subsequent recipients when high enough cell numbers were transplanted to provide sufficient coverage of the original complexity. These results demonstrate that ALL founder cells have similar engraftment potential and long-term self-renewal capacity in xenograft models, without evidence of clonal succession. Taken together, our data show that xenografted ALL samples consist of high numbers of cells able to establish the leukaemia and that each of these founder cells has a similar capability to drive the leukaemia.

**Figure 3:**
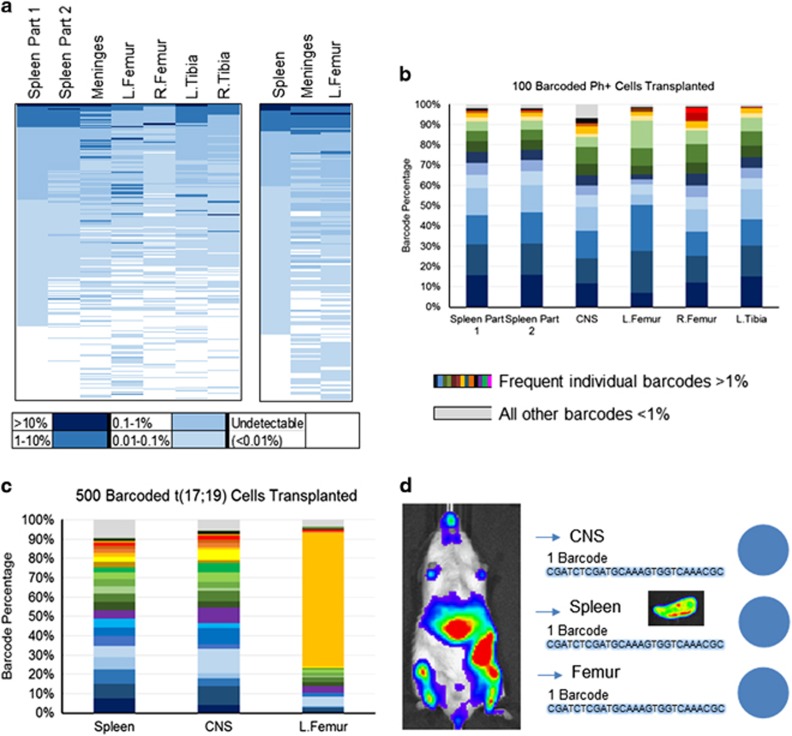
ALL founder cells have equal capacity to engraft different sites. (**a**) Heat map showing frequency of individual barcodes in different organs. Each heat map represents a single mouse. Thousand barcoded Ph+ (L4951) cells were transplanted. All transplant numbers represent the number of barcoded cells transplanted, which comprised ~10% of the total transplanted population. Spleens part 1 and 2 are different sections of the same spleen. (**b**, **c**) Graphs comparing barcode composition in different parts of the spleen with different bone marrow niches and CNS (meninges). Each coloured bar represents a single barcode with a frequency of at least 1% in at least one sample, light grey area shows all other barcodes. Colours correspond to the same barcode within each graph but not between different graphs. Hundred barcoded Ph+ (L4951) cells (**b**) or 500 barcoded t(17;19) cells (**c**) were transplanted. (**d**) Recovery of a single barcode from the injected femur, spleen and meninges following transplant of 10 barcoded Ph+ (L4951) cells. Images taken using IVIS spectrum (caliper) and produced using Living Image software.

To investigate the ability of different founder cells to engraft different organs, we collected samples from individual femurs, tibias and the meninges within the central nervous system (CNS) and compared their barcode compositions with the corresponding spleen. At low transplant doses, the vast majority of barcodes detected in the spleen were also found in the bone marrow and CNS samples and these different sites often, but not always, had similar compositions (Figures 3a and c and Supplementary Figure S3). Generally, common splenic barcodes had also engrafted other sites: of barcodes which comprised more than 1% of the labelled spleen populations across all samples, 100% were also detectable in paired CNS samples, 78% in the injected femur and 100% in the non-injected femur at frequencies above 0.01%. This demonstrates that all engrafting barcoded cells have equal capability to reconstitute the leukaemia in different environments. By transplanting as few as 10 labelled cells, we demonstrated directly that reconstitution of the leukaemia at all sites requires only a single clone (Figure 3d). Together, these data provide a picture of functional homogeneity of ALL blasts in the absence of external selection pressures, whereby all founder cells have the potential to contribute to the disease at all sites.

### Leukaemic composition can vary stochastically at different sites

**Figure 4:**
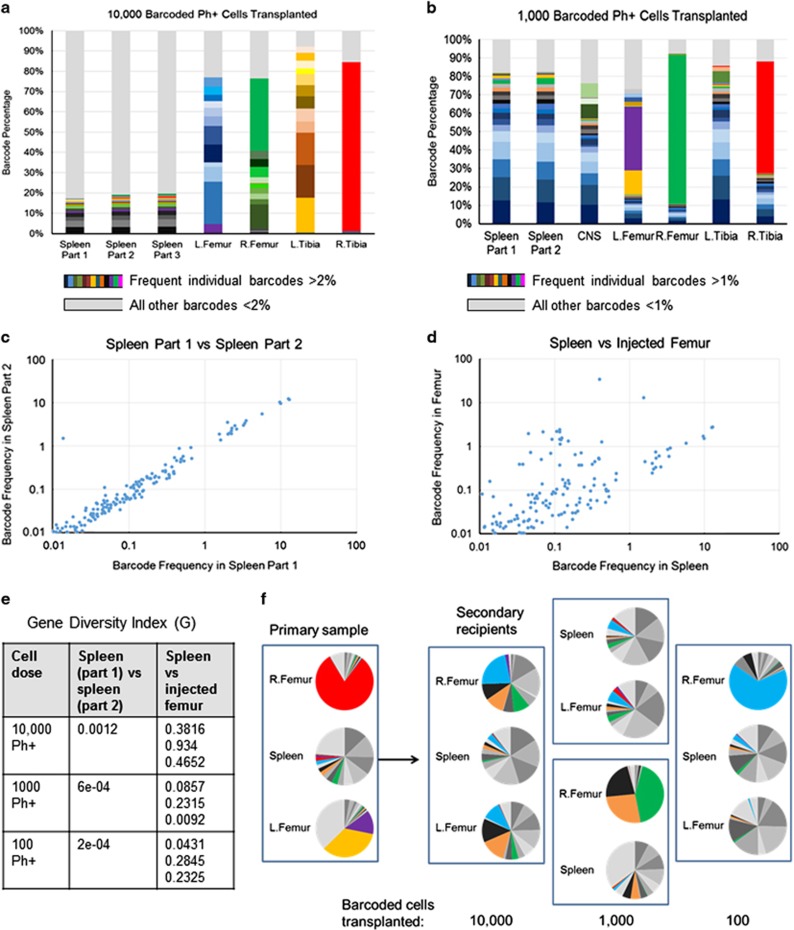
Spatial diversity of ALL founder clones at high transplant doses. (**a**, **b**) Graphs comparing barcode composition in different parts of the spleen with different bone marrow niches. Spleen parts 1, 2 and 3 are different sections of the same spleen. Each coloured bar represents a single barcode with a frequency of at least 2% in at least one sample, light grey area shows all other barcodes. Colours correspond to the same barcode within each graph but not between different graphs. Ten thousand (**a**) or 1000 (**b**) barcoded Ph+ (L4951) cells were transplanted, which comprised ~10% of the total transplanted population. (**c**, **d**) Graphs comparing frequency of individual barcodes in the spleen compared to a different part of the same spleen (**c**) or the injected femur (**d**) for a single mouse. Ten thousand barcoded Ph+ (L4951) cells were transplanted. (**e**) Table showing Nei’s Gene Diversity Index G, comparing different parts of the spleen and the spleen with the injected femur. Each number shows G for a single mouse. (**f**) Pie charts showing barcode composition in the spleen and femurs of a primary recipient (left box) compared to subsequent secondary recipients (right boxes) for Ph+ (L4951) sample. Individual blocks represent barcodes above 5% frequency in at least one sample. Selected dominant femoral barcodes are highlighted in the same colour across all samples.

To our surprise, we found some substantial differences in the barcode composition of different bones, which were particularly evident when we transplanted higher cell numbers, with each femur or tibia typically dominated by small numbers of barcoded founder populations in several different mice (Figures 4a and b and Supplementary Figure S4). This contrasted with the composition in different parts of the spleen, which was nearly identical (Figures 4c and d). To investigate this further, we quantified the levels of stochastic difference in barcode frequency, measured by Nei’s gene diversity index G^17^ (Figure 4e). We first measured G between different parts of the spleen, which confirmed that there is no subdivision, whereas the femurs showed drift away from the composition of the spleens, in particular at high transplant doses. These differences in the barcode composition of distinct sites could be caused by a selection process based on intrinsic growth advantages of specific founder cells in the given bone marrow environment. Alternatively, stochastic processes may direct barcode composition. In support of the latter, the dominant barcodes were never the same in the different bone marrow compartments. Furthermore, the most frequent barcodes in femurs of secondary transplants were different to those dominating in the primary mouse, even though the dominant primary founder populations were detectable in some secondary recipients at low frequencies (<0.1%) (Figure 4f and Supplementary Figure S5).

**Figure 5:**
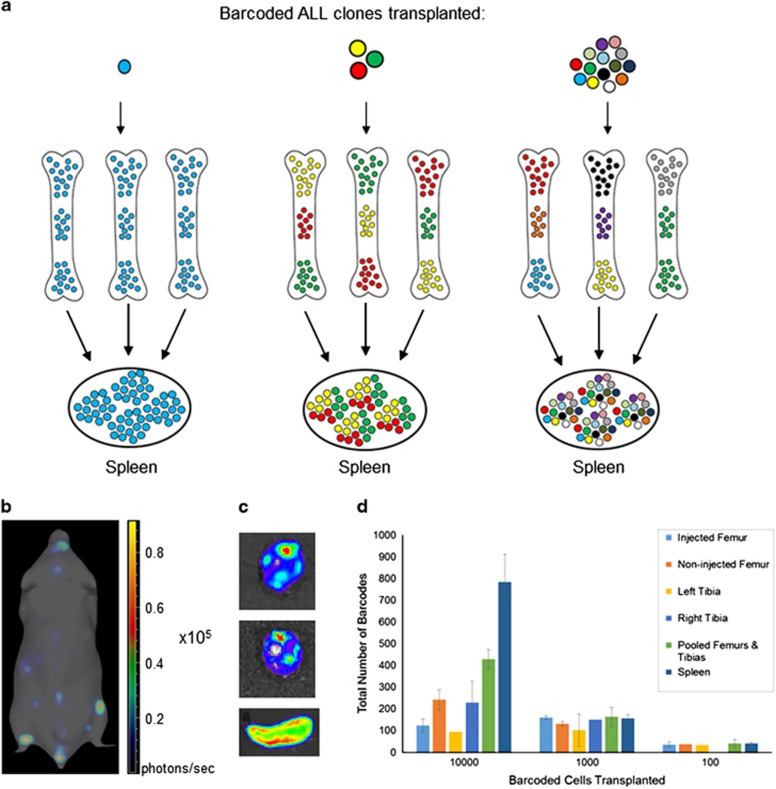
Focal growth of ALL founder clones. (**a**) Proposed model for growth of ALL founder clones. ALL cells grow focally in the bone marrow, leading to spatial diversity at high transplant doses. The spleen samples the whole blood supply so will be more representative of the full repertoire of engrafting cells. (**b**) Three-dimensional IVIS image showing luciferase-tagged xenografted high hyperdiploid ALL sample, produced using Living Image software. (**c**) IVIS images of calvarial bone marrow (top, middle) and spleen (bottom) from Ph+ (L4591) transplanted mice. Meninges were removed from calvaria before imaging. (**d**) Chart showing total number of recovered barcodes in the spleen, individual femurs and tibias, and pooling of all available femurs/tibias, of mice transplanted with varying doses of Ph+ sample. Error bars show standard deviation for up to three mice.

In summary, although all leukaemia propagating cells are functionally equipotent in the absence of external selective pressures, our results suggest a model whereby the tendency of cells to grow focally in restricted sites such as the bone marrow leads to stochastic spatial heterogeneity, similar to that of solid tumours.^12^ The spleen, through its action as a blood filtering system,^18^ will sample circulating blasts representing the full repertoire of engrafting barcoded founder cells in the mouse without spatial diversity (Figure 5a). In support of this, using imaging of luciferase-tagged ALL cells, we have previously shown evidence of local growth at different sites in the mouse, including in the long bones (Figure 5b).^15^ This was also evident in the calvarial bone marrow, in contrast to the spleen where the leukaemic cells were homogenously distributed (Figure 5c). Given that we have only looked at the murine tibias and femurs, which represent around 15% of the total bone marrow mass,^19^ this model suggests that the overall composition of the whole mouse bone marrow will be similar to that of the spleen. In support of this, the number of recovered barcodes increased when the bone marrow samples were pooled rather than considered as individual bones (Figure 5d).

## Discussion

Our results have demonstrated that xenografted high-risk ALL samples consist of high numbers of founder cells and that a multitude of these founder cells maintain the leukaemia in primary and secondary mice. These data support and extend our previous work, which assessed engraftment potential of ALL blasts based on phenotype^11^ and limiting dilution analysis.^10^ Here we now show that not only are many cells able to engraft (high abundance of engrafting founder cells), but that these founder cells have similar ability to reconstitute the complete leukaemia in different organs in the mouse—each cell contributes equally to the leukaemia without evidence of dominance or succession (equipotency). Our work complements previous data showing that xenograft models can support the growth of genetically distinct subclones by studying clonal diversity at a higher resolution.^3, 6^ Given that we have used samples that have previously been passaged through mice, we cannot exclude the possibility that primary ALL samples contain subclones with different properties, which are unable to engraft mice. However, it has previously been shown that primary samples also contain high frequencies of engrafting cells^10^ and that xenografts can recapitulate the complexity of the original sample in high-risk patients.^4, 7^ The ability of all blasts to engraft different organs is particularly important for treatment of the CNS compartment, as any CNS-directed therapy will need to eradicate all leukaemic cells rather than specific CNS engrafting subclones. This confirms the findings of Williams *et al*,^20^ who recently demonstrated that CNS infiltration is a generic property of ALL blasts.

Importantly and to our initial surprise, we show that leukaemia can exhibit spatial diversity, with different barcode composition emerging in different bone marrow sites. It is well established that solid tumours can vary in clonal composition, both within the primary tumour and in comparison to sites of metastasis.^12^ Our results suggest that this also applies to leukaemia. We propose that this is related to the tendency of leukaemias to grow focally at different sites in the mouse, whereas the spleen acts to sample the total blood volume so is more representative of all engrafting founder cells. The dominance of certain founder populations found in some bone marrow sites does not appear to be due to hard-wired properties of particular cells, as the identity of the dominant barcodes was not preserved. Instead, we propose this occurs due to stochastic or chance-based processes, which originate extrinsic to the cell. These could include founder effects, whereby the first cells to arrive at a particular site are able to establish an advantage, or exposure to particular niches, which may provide a growth advantage. The fact that the dominance occurred in the marrow of the long bones, but rarely the spleen, suggests that the microenvironment plays a key part. It has been established that leukaemia cells are able to modulate the bone marrow environment using tunnelling nanotubules^21^ and that niche remodelling in response to therapy can lead to drug resistance.^22^ Furthermore, a recent study demonstrates that *in vivo* microenvironments can induce a reversible, dormant, drug-resistant phenotype in leukaemic blasts.^23^ Because of insufficient coverage of rare clones, our system will not detect dormancy at the founder cell level following transplantation, however, it is likely that individual daughter cells derived from the different founders will nest into the endosteal bone marrow niche^23^ where they become quiescent. The pattern we observe in the bone marrow also has some resemblance to that found following transplant of barcoded haematopoietic stem cells, which become asymmetrically distributed among different skeletal niches.^24^ This spatial heterogeneity has implications for studies examining clonal diversity in patient samples, as the site which material is taken from could impact the clonal heterogeneity observed. It is sometimes assumed that dominant subclones have acquired advantageous mutations, and that studying their genetics can identify new therapeutic targets. Our results show that caution should be taken when interpreting these results. They also imply that chance can play a significant role in disease evolution and relapse, as response to treatment may depend on whether subclones with potential to develop resistance make it into protective niches or areas with poor pharmacokinetics before the start of therapy. This apparent capacity for clonal dominance without selection pressure also needs to be considered for murine *in vivo* functional genomic screening approaches, as dominance can occur even when cells receive apparently non-functional constructs. This effect can be limited by taking spleen samples rather than bone marrow, transplanting high cell numbers (>100 000) and validating results in multiple mice.

**Figure 6:**
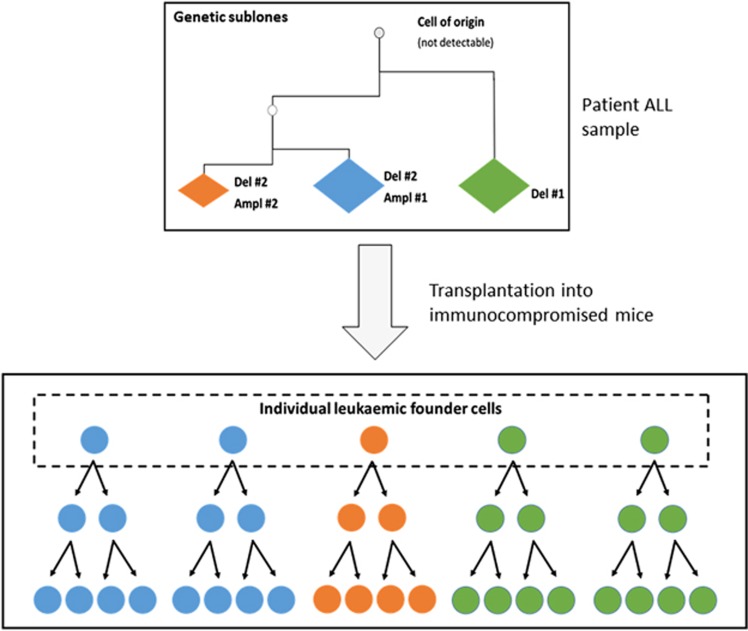
Suggested integrated model for genetic subclones and barcoded founder cells. Patient ALL samples consist of multiple genetically defined subclones (rhombus shapes in top panel), each of which will contain high numbers of individual cells. Following transplantation into immunocompromised mice, individual blasts from each genetic subclone will engraft and propagate the leukaemia, leading to a multitude of cells with propagating capacity. Each individual blast will therefore be a potential unit of selection.

Taken together, our findings support the stochastic stem cell model in ALL. Although we cannot formally rule out a shallow stem cell hierarchy with a high frequency of leukaemia-initiating cells, the combination of the data in this study and our previous work demonstrating equal leukaemic-initiating capacity in blasts of all immunophenotypes^10^ strongly supports the idea that stem cell potential is a generic property of a high proportion of ALL blasts. This does not necessarily mean that each of these cells actually drives the leukaemia in every given situation, merely that they all have the capability to do so if required. As Till and McCulloch predicted,^25^ there is no clearly defined stem cell hierarchy in the lymphoid lineage and lymphoid cells maintain their ability for clonal expansion throughout maturation. Malignant cells hijack the pre-B-cell and B-cell receptor checkpoints, which regulate normal lymphoid development to suppress negative selection and gain the ability for uncontrolled clonal expansion.^26^ Our data shows that this process results in a large number of potential units of clonal selection and evolution. Our results complement the previously described model of genetic subclonal architecture in ALL,^3, 6^ as each genetic subclone will consist of a multitude of leukaemia propagating cells (Figure 6). Despite the abundance of units for selection, most ALL genomes contain fewer than 10–20 mutations, most probably due to the relatively low genomic instability of ALL.^27^ The prevalent model of tumour heterogeneity based purely on these subclonal genetic differences cannot therefore fully explain the evolution and development of ALL, especially in the case of relapse. Our data demonstrate that this process will also have a stochastic component. The functional status of a cell will depend on a multitude of factors beyond genetics, including, tumour microenvironment, epigenetics and cellular signalling networks.^28^ Indeed, recent work has suggested that crosstalk between leukaemic blasts and the microenvironment may lead to epigenetic reprogramming of the blasts under chemotherapy.^29^ Changes such as these will act in tandem with intrinsic subclonal differences, which may only become functionally relevant under selection pressures, to determine the evolution of the disease. Successful treatment strategies will therefore need to ensure eradication of all the leukaemic blasts, while also considering targeting any resistant clones which emerge.

## Figures and Tables

**Table 1:** Engrafting cell frequencies in primograft ALL samples

*Sample*	*Maximum transduction %*	*Total cells injected*	*Barcoded cells*	*Time to harvest*	*Average barcodes recovered (number of mice)*	*L-IC frequency*
L4951 Ph+	10	100 000+900 000 unlabelled	10 000	69 days	662 (3)	1:15
		100 000	10 000	71 days	784 (3)	1:13
		10000	1000	83 days	155 (3)	1:6
		1000	100	90 days	41 (3)	1:2
L4967 Ph+	5	100 000	5000	42 days	798 (2)	1:6
P929 t(4;11)	1	150 000	1500	78 days	52 (2)	1:29
L707 t(17;19)	5	100 000	5000	36 days	705 (2)	1:7
	5	10 000	500	37 days	56 (2)	1:9

Frequencies were calculated by the following formula: (total cells transplanted) × (transduction %)/barcodes recovered from spleen.
