# Supplementary material for: Abundant and equipotent founder cells establish and maintain acute lymphoblastic leukaemia
Source: Leukemia. 2017 May 26;31(12):2577–86. doi: 10.1038/leu.2017.140 (PMC5558874; doi:10.1038/leu.2017.140)
Supplement: Supplementary Material [file leu2017140x1.docx]

**Supplemental Methods**

**Barcode library construction**

Barcode oligonucleotides (Forward: 5’-GATCCNNATCNNGATSSAAANNGGTNNAACNNTG TACAACGACGTCCAGC-3’, reverse: 5’-TCGAGCTGGACGTCGTTGTACANNGTTNNACCN NTTTSSATCNNGATNN G-3’) were resuspended at 200 µM in water and mixed at equal volumes with 5x annealing buffer (0.5M Tris HCl pH 7.4, 0.35M MgCl_2_). The oligos were annealed by heating to 95^O^C for 5 min and cooling to room temperature. The annealed linker was cloned into pENTR1A Gateway entry vector (Life Technologies) digested with BamHI and XhoI at 1:20 molar ratio. The vector was transformed into Max Efficiency DH5α bacteria (Life Technologies) and plasmid collected using HiSpeed Maxiprep Kit (Qiagen). The barcode region was subsequently transferred into a lentiviral vector, pSLIEW^1^, into which we had inserted a Gateway Destination cassette at the BamHI site 5’ to the IRES2 sequence, using LR clonase II (Life Technologies). Two separate 10 µl reactions were performed using 100 ng of entry clone and 150 ng of destination vector. The whole reaction mixture was transformed into Max Efficiency Stbl2 (Life Technologies) at 1 µl per 50 µl of bacteria. The transformations were pooled, grown at 30^O^C for 16 h and barcode library collected using EndoFree Maxiprep Kit (Qiagen). The complexity of the library was validated by Illumina MiSeq sequencing.

**Patient samples**

Patient derived material was collected as part of the initial diagnostic investigation of patients. It was collected, stored and used with written informed consent according to approvals given by the local institutional review boards and the Declaration of Helsinki. Samples were retrieved from Newcastle Haematological BioBank under the generic BioBank approval given by the Newcastle & North Tyneside Ethics Committee (REC reference number: 07/H0906/109+5).

**Lentiviral transduction & mouse work**

Primograft material was obtained by serially passaging primary leukemic blasts in NSG mice^2^. Samples L4951, L4967 and P929 were passaged once prior to transduction, L707 was passaged three times. Virus production and transduction of primograft material was performed as described previously^1^. To reduce the risk of multiple lentiviral integrations, we limited transduction of primografts to approximately 10%^3, 4^. Transduction efficiencies were analysed by measuring GFP expression using a FACS Calibur (BD) and the transduced material was injected into the left femurs of 8-12 week old male NSG mice. Engraftment was monitored by imaging using the IVIS Spectrum (Caliper Life Sciences). Mice were kept until they began to exhibit clinical signs which necessitated humane killing. All work was conducted in accordance with the Home Office Project Licence PPL60/4552.

**Sample collection and preparation**

All spleens were substantially enlarged and samples were collected by homogenising material through a cell strainer with phosphate buffered saline (PBS). Bone marrow samples were collected by flushing the lumens with PBS. Meningeal samples were collected by gently vortexing the brains to detach the meninges and combining with material scraped from the deep surface of the calvarium. Genomic DNA was extracted using a DNeasy Blood & Tissue kit (Qiagen).

**Barcode PCR amplification and sequencing**

Barcode regions were amplified from plasmid or genomic DNA by 20 cycles of PCR using Phusion Hot Start II DNA Polymerase (Thermo Scientific) and primers specific to the barcode region (forward: AATTCAGTCGACTGGATCC, reverse: CATATAGACAAACGCACACC). To obtain as accurate an estimate as possible of the number of engrafting blasts, it is important to ensure rare barcodes are maintained at reasonable coverage throughout the PCR amplification and sequencing analysis. The highest number of cells transplanted for any sample was 100,000, so based on a transduction rate of approximately 10% a maximum of 10,000 barcodes were expected in these samples. We therefore performed sufficient PCRs to ensure that barcodes representing 0.01% of the total would be present with at least 25-fold coverage. A maximum of 825 ng of DNA was added to each PCR reaction, with multiple reactions performed as required. Reactions were pooled and purified using Qiaquick PCR Purification Kit (Qiagen). Illumina sequencing primers (Forward: AATGATACGGCGACCACCGAGATCTACACTCTTT CCCTACACGACGCTCTTCCGATCTNNNNAATTCAGTCGACTGGATCC, reverse: CAAGCAGAAGACGGCATACGAGATNNNNNNGTGACTGGAGTTCAGACGTGTGCTCTTCCGATCTGAGCTGGACGTCGTTGTAC, N in reverse represent multiplex index tag) were added using a further 20 PCR cycles. A stretch of 4 random nucleotides was included after the Illumina sequencing primer binding site to increase initial complexity to aid with cluster calling ^5^. Products were gel extracted, pooled and sequenced using a 50 bp PE Illumina MiSeq run.

**Bioinformatics & data analysis**

Sequencing data was analysed using a custom python script (provided as supplemental attachment) to extract barcode sequences and frequencies from each read. The script provides counts for barcodes with the correct series of fixed triplets that had Illumina quality scores above 20. To distinguish between real barcodes and those arising due to errors in PCR amplification or sequencing, we required a cut-off level whereby low frequency reads would be removed. The PCR amplification was designed to give sufficient coverage to barcodes representing above 0.01% of the total population, so we could not be confident that barcodes below this level were real. Based on this we removed barcodes comprising less than 0.01% of the total reads. The use of this threshold means that the engrafting cell frequencies (see Table 1) are likely to be more accurate at lower transplant doses, where there is a clearer distinction between real barcodes at higher frequencies and artefacts at lower frequencies; as opposed to higher transplant doses where rarer barcodes may be at frequencies which overlap with sequencing artefacts. Barcodes above the 0.01% level were also manually removed if they either had single base changes from high frequency barcodes within that same sample, or were identical to high frequency barcodes from different mouse samples in the same sequencing run, suggesting incorrect assignment during demultiplexing ^6^. Sequences which did not match the expected fixed triplets were processed to create a list of ‘rejected’ barcodes, which had the correct vector sequence leading up to the barcode, but contained mismatches or insertions/deletions within the barcode region. To avoid discarding important barcodes which did not match the expected fixed sequence due to errors in the library cloning process, rejected barcodes that were i) present in samples at high frequencies (above 10% of the total in 1 organ), ii) present in the list of rejected barcodes from the original library and iii) did not match any ‘real’ barcodes when the error in the fixed sequence was corrected, were included in the final analysis.

**Measurement of gene diversity index (G)**

Estimating G from barcode tag count Data:

We follow Nei (1973)^7^ in investigating diversity of barcode tags by considering the identity of a pair of tags sampled within a population or populations. The estimation procedures are similar to those of F statistics for population genetics, but these are described in terms of heterozygosity of individuals and inbreeding and this is not a suitable framework for tracking barcode tagged cells.

The probability that a randomly sampled pair of cells carry the tag, for a population of cells with $k$ tags is given by

$$D_{a}=\sum_{i=1}^{k} p_{i}^{2},$$

where the relative frequency of tag $i$ is $p_{i}$

If we consider another population which has undergone drift with respect to the original population, then we can write down the expected probability of identity as

$$D_{s}=G\sum_{i=1}^{k} p_{i}+(1-G)\sum_{i=1}^{k} p_{i}^{2}.$$

Here $G$ is a measure of the increase in probability that a pair of tags are the same. If some tags drift upwards in frequency and some downwards, then the probability that a random pair are identical increases.

For random drift for a constant population size of $N$ over $t$ generations, $G=1-(1-1/N)^{t}\approx1-\exp(-t/N)$.

Following Nei, $G$ is estimated by $\frac{D_{s}-D_{a}}{1-D_{a}}$.

In the next section we describe how to estimate $G$ from data. We call this estimator $\hat{G}$.

Initial Transplantation:

Consider estimation after the initial transplantation, and assume that we can get an accurate assessment (without error) of the frequencies of tagged cells in any population of cells. We shall consider the error introduced by genotyping in a later section.

Before transplantation, the probability that a pair of tagged cells sampled at random (with replacement) are identical is given by $\sum_{i=1}^{k} 1/k^{2}=1/k$, where $k$ is the number of different tags.

We want to measure how much the tagged cell frequencies have changed. We consider again the probability of identity for a pair of tags relative to the change under no drift. This can be written as

$$\hat{G}=\frac{\sum_{i=1}^{k} (p_{i}-1/k)^{2}}{\sum_{i=1}^{k} (1-1/k)/k}=k/(k-1)\sum_{i=1}^{k} (p_{i}-1/k)^{2}.$$

Drift away from a Population of Tags:

When we consider drift away from a baseline population with relative tag frequencies $b_{i}$, then we use the following formula.

$$\hat{G}=\frac{\sum_{i=1}^{k} (p_{i}-b_{i})^{2}}{\sum_{i=1}^{k} b_{i}(1-b_{i})}$$

Errors in Allele Frequency Estimation:

For our tag data we do not observe $p_{i}$, instead we get counts for each tag. These are observed with random errors. If we assume that the counts are poisson distributed with mean equal to $np_{i}$ for tag $i$, where $n$ is the total count, then we can judge the relative sizes of errors.

The standard error of the estimate of $p_{i}$ is $\sqrt{p/n}$ and the change from drift is of the order of $\sqrt{Gp}$. For small $p$, we can show that, under the poisson count model, provided $G>>1/n$ then errors due to drift will overwhelm errors due to count data.

For small $n$, we can improve our estimates of $G$ by using the formula

$$\hat{G}=\frac{\sum_{=1}^{k} (p_{i}-b_{i})^{2}}{\sum_{i=1}^{k} b_{i}(1-b_{i})}-\frac{1}{n}-\frac{1}{n_{b}}$$

where $n_{b}$ is the total count for the baseline tag frequencies.

R code for the estimation of G is at the web site <https://github.com/ijwilson/diversit-tag>.

**References**

1. Bomken S, Buechler L, Rehe K, Ponthan F, Elder A, Blair H*, et al.* Lentiviral marking of patient-derived acute lymphoblastic leukaemic cells allows in vivo tracking of disease progression. *Leukemia* 2013 Mar; **27**(3)**:** 718-721.

2. Rehe K, Wilson K, Bomken S, Williamson D, Irving J, den Boer ML*, et al.* Acute B lymphoblastic leukaemia-propagating cells are present at high frequency in diverse lymphoblast populations. *EMBO molecular medicine* 2013 Jan; **5**(1)**:** 38-51.

3. Fehse B, Kustikova OS, Bubenheim M, Baum C. Pois(s)on--it's a question of dose. *Gene therapy* 2004 Jun; **11**(11)**:** 879-881.

4. Charrier S, Ferrand M, Zerbato M, Precigout G, Viornery A, Bucher-Laurent S*, et al.* Quantification of lentiviral vector copy numbers in individual hematopoietic colony-forming cells shows vector dose-dependent effects on the frequency and level of transduction. *Gene therapy* 2011 May; **18**(5)**:** 479-487.

5. Krueger F, Andrews SR, Osborne CS. Large scale loss of data in low-diversity illumina sequencing libraries can be recovered by deferred cluster calling. *PloS one* 2011; **6**(1)**:** e16607.

6. Kircher M, Sawyer S, Meyer M. Double indexing overcomes inaccuracies in multiplex sequencing on the Illumina platform. *Nucleic acids research* 2012 Jan; **40**(1)**:** e3.

7. Nei M. Analysis of gene diversity in subdivided populations. *Proc Natl Acad Sci U S A* 1973 Dec; **70**(12)**:** 3321-3323.

**Supplemental Figures**


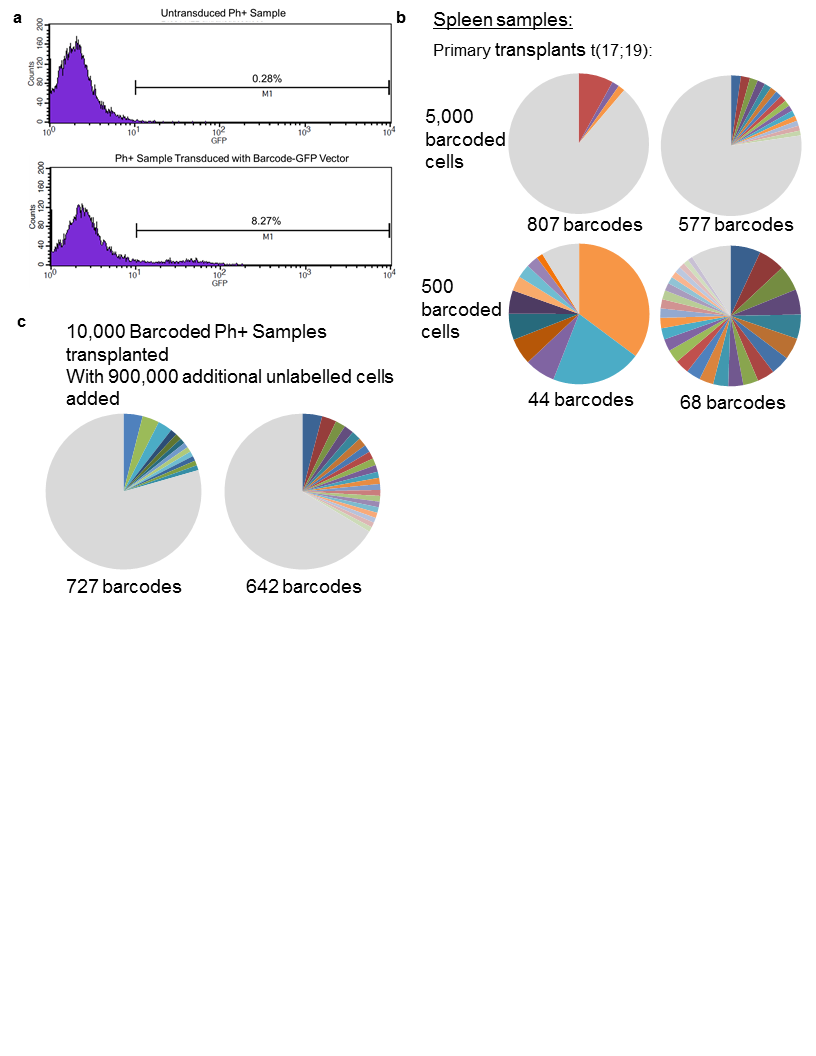


**Figure S1.** (**a**) Example histogram showing transduction rate of Ph+ sample transduced with barcode virus (bottom) compared to unstransduced control (top). (**b**) Composition of barcoded population at varying transplant doses for t(17;19) ALL sample. Transplant numbers represent the number of barcoded cells transplanted, which comprised approximately 5% of the total transplanted population. Each pie chart shows barcode composition in spleen sample from a single mouse. Coloured segments each represent a unique barcode comprising >1% of the total, light grey segment shows all other barcodes <1%. Colours do not represent the exact same barcode on different pie charts. (**c**) Composition of barcoded population following transplant of 10,000 Barcoded Ph+ cells (as in Figure 1b, top panel), together with an additional 900,000 untransduced cells. Each pie chart shows barcode composition in spleen sample from a single mouse. Coloured segments each represent a unique barcode comprising >1% of the total, light grey segment shows all other barcodes <1%. Colours do not represent the exact same barcode on different pie charts.


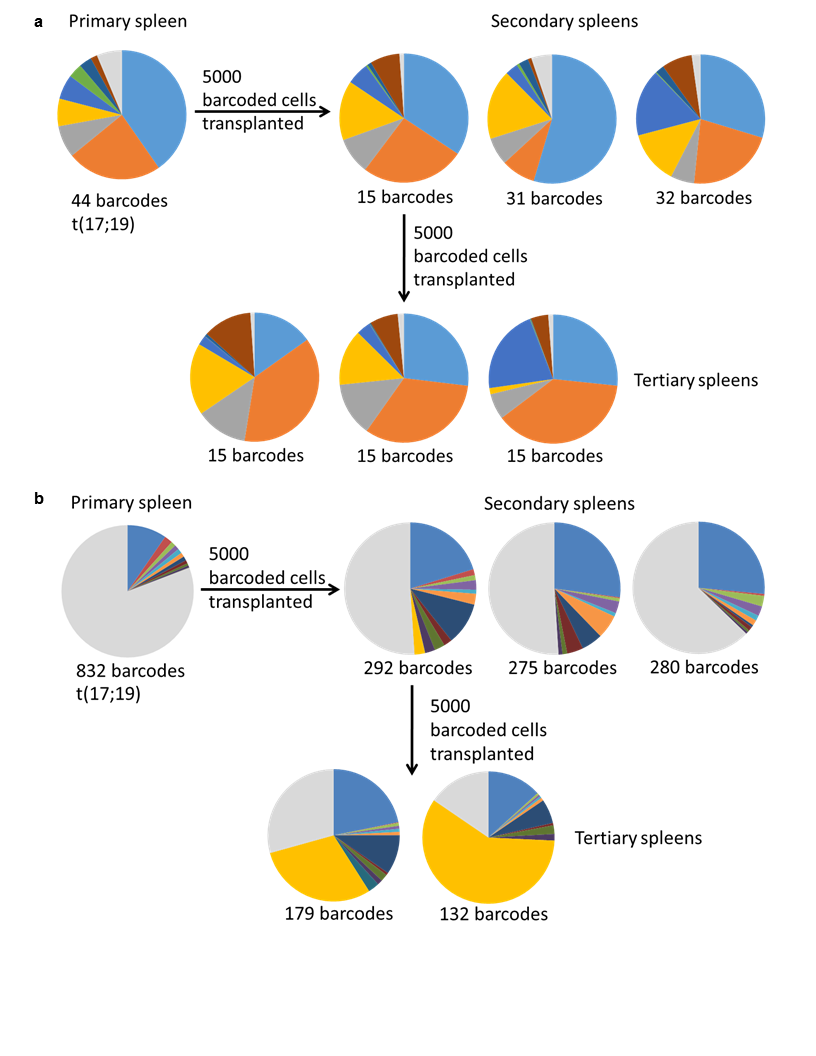


**Figure S2.** (**a,b**) Secondary and tertiary transplants for t(17;19) L707 ALL sample. Primary samples were spleens from initial transplants of 500 (**a**) or 5,000 (**b**) barcoded cells. Each pie chart is a single mouse spleen. Numbers below each chart represent the total number of recovered barcodes in that sample which were also present in the parent sample. Colours correspond to the same barcode for each mouse within each transplant set (e.g. within panel 2a), but not between (e.g. panel 2a compared to 2b). Panel (b) potentially shows enrichment of a previously minor clone in both tertiary mice, although it is unclear whether this is related to acquired properties of this clone or occurred by chance.


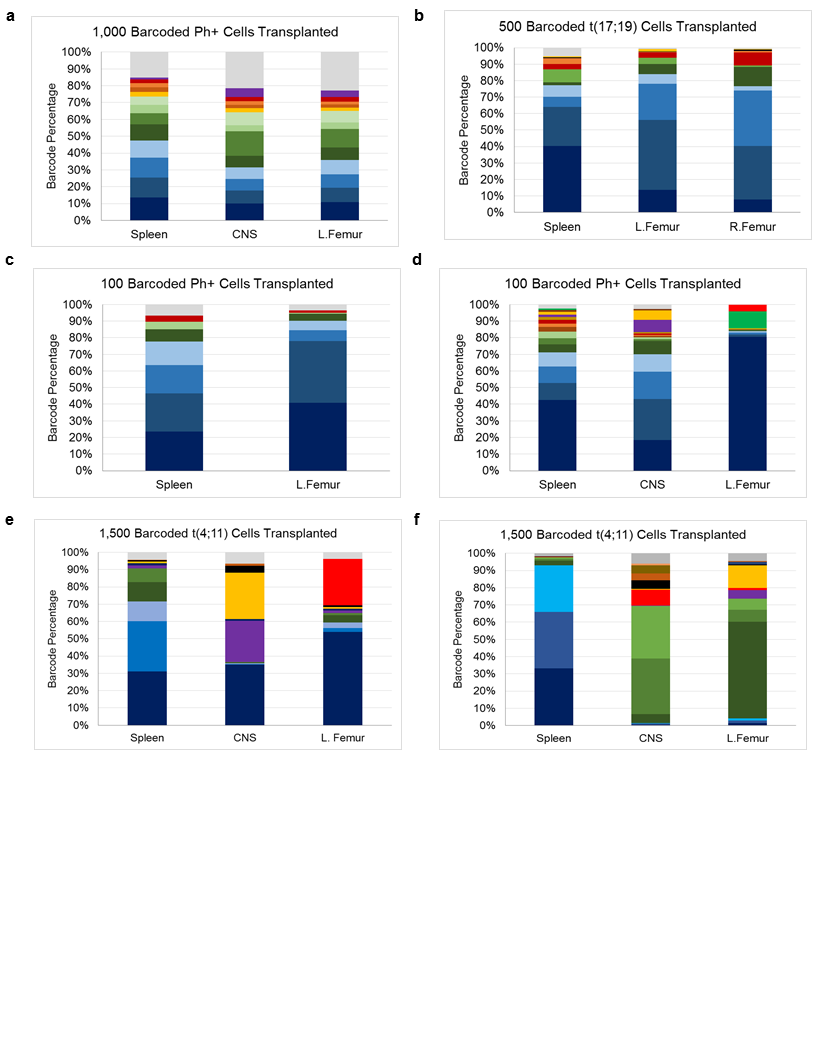


**Figure S3.** (**a-f**) Graphs comparing barcode composition in different parts of the spleen with different bone marrow niches and CNS (meninges) for Ph+ (L4951) (**a,c,d**), t(17;19) (**b**) and t(4;11) samples at low transplant doses. Each coloured bar represents a single barcode with a frequency of at least 1% in at least one sample, light grey area shows all other barcodes. Colours correspond to the same barcode within each graph but not between different graphs Samples were transduced at a rate of approximately 10% (Ph+), 5% (t(17;19)) or 1% (t(4;11)), graph titles show the number of barcoded cells transplanted.


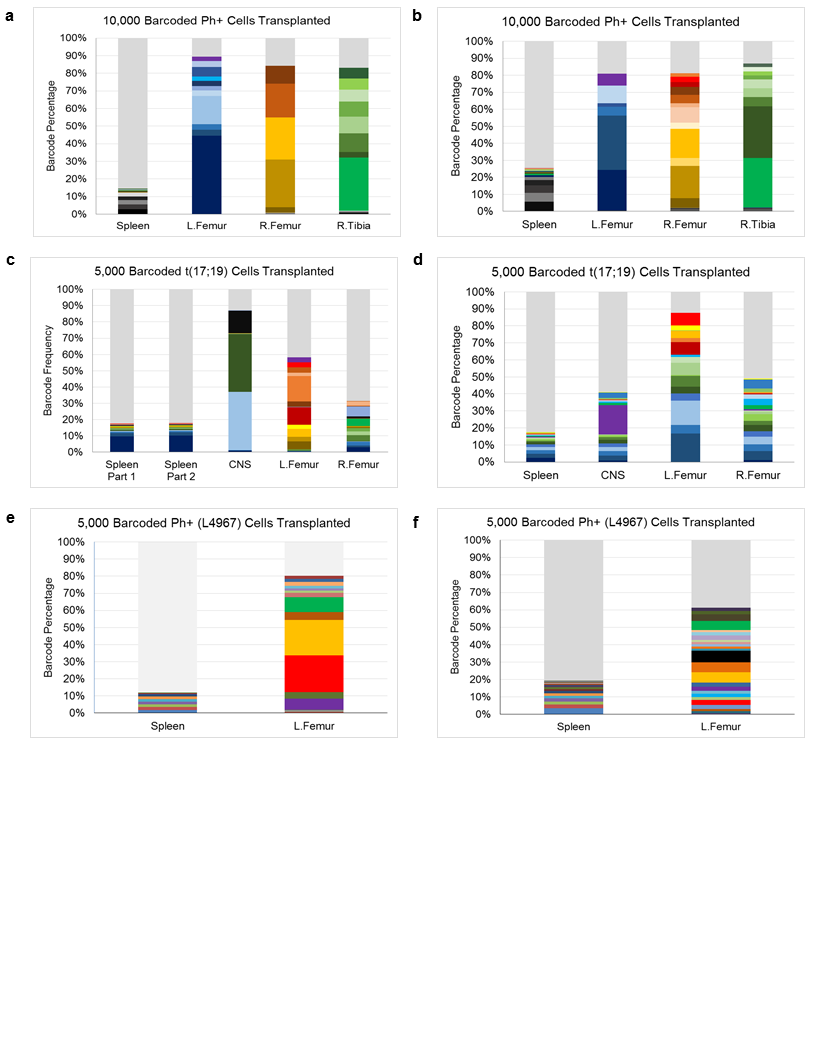


**Figure S4.** (**a-f**) Graphs comparing barcode composition in different parts of the spleen with different bone marrow niches and CNS (meninges) for Ph+ (L4951) (**a,b**), t(17;19) (**c,d**) and Ph+ (L4967) (**e**,**f**) samples at high transplant doses. Each coloured bar represents a single barcode with a frequency of at least 2% in at least one sample, light grey area shows all other barcodes. Colours correspond to the same barcode within each graph but not between different graphs Samples were transduced at a rate of approximately 10% (Ph+ L4951) or 5% (t(17;19), Ph+ L4967), graph titles show the number of barcoded cells transplanted.


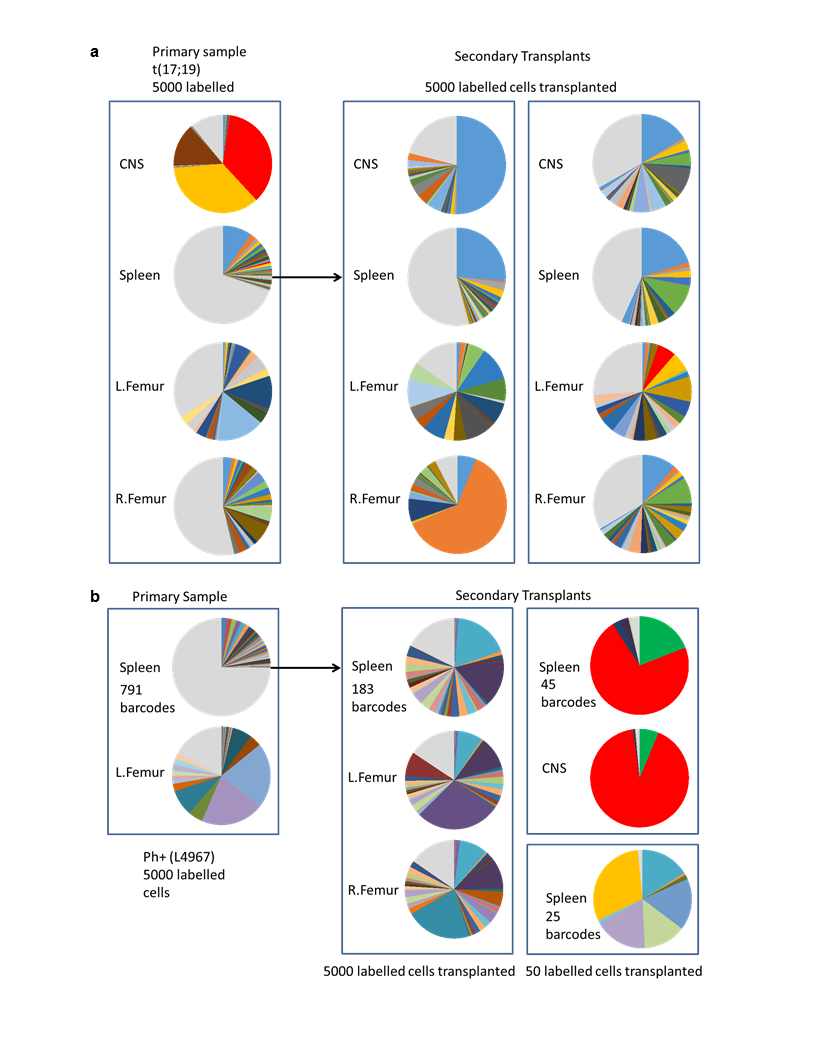


**Figure S5.** (**a,b**) Composition of femurs, spleens and bone marrow in secondary transplants of t(17;19) L707 (**a**) and Ph+ L4967 (**b**) ALL samples. Primary samples were spleens from mice with initial transplants of 5,000 barcoded cells which were transplanted into secondary recipients at the stated doses. Each box shows a single mouse. Colours correspond to the same barcode for each mouse within each transplant set (e.g. within panel 2a), but not between (e.g. panel 2a compared to 2b).
